# Complete genome and plasmid sequences of *Moraxella* sp. strains K02 and K23, closely related to *M. osloensis*, isolated from freshwaters

**DOI:** 10.1128/mra.00619-24

**Published:** 2024-09-27

**Authors:** Ryoka Wakabayashi, Takeshi Naganuma, Ryosuke Nakai

**Affiliations:** 1 Graduate School of Integrated Sciences for Life, Hiroshima University, Higashi-Hiroshima, Japan; 2 Bioproduction Research Institute, National Institute of Advanced Industrial Science and Technology (AIST), Sapporo, Japan; Department of Biology, Queens College, Queens, New York, USA

**Keywords:** *Moraxella osloensis*, cosmopolitan, environmental strain

## Abstract

*Moraxella osloensis* is a cosmopolitan bacterium, ranging from human body to diverse environments. Only 2 of the 11 complete genomes so far are from non-clinical strains. Here, we present the complete genome and plasmid sequences of two freshwater isolates. The data will provide insights into the cosmopolitanism of this species.

## ANNOUNCEMENT

*Moraxella osloensis* of the phylum *Pseudomonadota* is known to kill slugs (1) and cause laundry malodor (2). *M. osloensis* occurs in the human body (3) and various environments. Only 2 of the 11 complete genomes are from non-clinical samples (https://www.ncbi.nlm.nih.gov/datasets/genome/?taxon=34062, accessed August 2024). Here, we add the complete genome and plasmid sequences of *Moraxella* sp. strains K02 and K23, closely related to *M. osloensis*, from freshwaters that may help elucidate the cosmopolitanism of this species.

Strains K02 and K23 were isolated from streams in western Japan (31.23 N, 130.61 E, and 35.16 N, 132.84 E, respectively) and were stored in the dark at room temperature until isolation. Portions of the samples were inoculated onto NSY agar medium (4) and incubated at room temperature for several days. Small colonies were picked and transferred to fresh NSY agar plates for pure isolation.

For genome sequencing, strains K02 and K23 were cultured in R2A broth at room temperature for 1 week. DNAs were extracted from the culture samples using the Qiagen MagAttract HMW DNA Kit. After DNA shearing to 10–20-kbp fragments using g-TUBE (Covaris), a sequence library was prepared for each strain using the PacBio SMRTbell gDNA Sample Amplification Kit and SMRTbell Express Template Prep Kit 2.0 following the manufacturer’s protocol. No size selection was performed. The libraries were sequenced on a PacBio Revio with the corresponding polymerase kit. Default settings were used for all data processes. HiFi reads were obtained by removing overhang adapter sequences using SMRT Link (v.13.0.0.207600), creating aligned consensus sequences, and removing reads with an average quality per read of less than 20. Ultra-LowPCR adapter removal from HiFi reads was performed using lima (ver. 2.7.1), followed by PCR duplicate read removal using pbmarkdup (ver. 1.0.3). Reads of 1,000 bp or less were also removed using Filtlong (ver. 0.2.1). Finally, 33,818 and 39,453 HiFi reads with average lengths of 7,357 and 6,298 bp (total sequence lengths of 248,795,498 and 248,455,088 bp) for K02 and K23, respectively, were obtained. The reads were assembled with Flye ver. 0.2.1 (5), and the overlapping ends of circularized contigs were identified and trimmed with Flye. The assembly completeness was assessed with Bandage ver. 0.8.1 (6) and CheckM2 ver. 1.0.1 (7). The chromosomes and plasmids were annotated with DFAST ver. 1.2.11 (8). The chromosomes were rotated to start at *dnaA* using DFAST option. To identify the full-length 16S rRNA gene sequence, we conducted a BLASTn search against the NCBI database (accessed on April 12, 2024).

The single chromosomal contigs of K02 and K23 were 2,515,657 and 2,477,111 bp, respectively (Table 1). The K02 chromosome contains 2,228 coding sequences (CDSs), 12 rRNA genes, 48 tRNA genes, and 1 CRISPR gene. The K23 chromosome contains 2,119 CDSs, 12 rRNA genes, 47 tRNA genes, and 2 CRISPR genes. Strains K02 and K23 exhibit high 16S rRNA gene sequence identities, 99.80% and 99.08%, and high chromosomal average nucleotide identity (ANI) values (9), 95.03% and 94.94%, respectively, to the type strain *Moraxella osloensis* CCUG350 (accession no. CP014238). The chromosomal ANI between K02 and K23 is 94.94%.

**TABLE 1:** Genome and plasmid sequences of two *Moraxella* strains K02 and K23

Strain	Contig	Chromosome/plasmid (accession number)	bp	G+C%	CDS	Coverage
K02	1	Chromosome (AP031590)	2,515,657	43.8	2,228	83
	2	K02p1 (AP031591)	78,262	39.1	76	66
Total fold coverage	3	K02p2 (AP031592)	69,683	40.8	61	72
90.2	4	K02p3 (AP031593)	46,393	39.9	48	59
Raw read N50	5	K02p4 (AP031594)	26,510	42.4	27	24
2,515,657	6	^*a*^	19,161	35.9	21	20
	7	K02p5 (AP036013)	2,380	34.7	2	155
K23	1	Chromosome (AP031595)	2,477,111	43.8	2,119	83
	2^*b*^	K23p1 (AP031596)	79,031	40.2	81	50
Total fold coverage	3	K23p2 (AP031597)	49,559	40.5	48	46
90.6	4^*b*^	K23p3 (AP031598)	49,233	41.1	45	76
Raw read N50	5	K23p4 (AP031599)	32,073	46.4	27	106
2,477,111	6	K23p5 (AP031600)	16,084	38.1	15	274
	7	K23p6 (AP031601)	13,890	37.2	12	65
	8	K23p7 (AP031602)	12,151	34.0	11	52
	9	K23p8 (AP031603)	11,740	39.6	8	75

^*a*^ K02’s contig 6 is linear and completely matches with a part of K02’s contig 4.

^*b*^ K23’s contigs 2 and 4 (K23p1 and K23p3) might form a single plasmid, which would be regarded as too large (128,590 bp) for a plasmid and thus was divided.

## Data Availability

The K02 and K23 genome sequences were deposited in the DDBJ/ENA/GenBank database under accession numbers AP031590–AP031603 and AP036013 (BioProject PRJDB18031; BioSample SAMD00772879–SAMD00772880; DDBJ Sequence Read Archive DRA018602).
